# Nervous Diseases

**Published:** 1905-11-25

**Authors:** 


### PROGRESS IN MEDICINE AND SURGERY.

### NERVOUS DISEASES.

(*Continued from page* 119.)

**Some Spinal Paralyses.**—The so-called "primary" spastic paralysis in adults, even in cases which have exhibited a fairly uniform clinical picture has been found post-mortem to be associated with such different conditions as transverse myelitis, cerebral tumour, multiple sclerosis, etc., and the existence of a primary systemic degeneration of the pyramidal tracts is questionable. In many cases a history of syphilis is obtained, and Osler expresses the opinion that most instances of such paralysis not due to slow compression of the cord are associated with this disease. Under the title of "Syphilitic Spinal Paralysis" Erb has differentiated a group of cases, of which the main feature is a spastic paralysis, although there is evidence of more or less involvement of other tracts in the cord besides the pyramidal fibres. Erb describes the following among the symptoms observed: A very decided spastic gait with the usual features of stiff, closely approximated legs and dragged feet; enormously exaggerated deep reflexes with patellar and ankle clonus and usually Babinski's sign, an extensor instead of a flexor plantar response; muscular weakness or paresis; rigidity, often slight in comparison with the spastic gait and excessive reflexes; weakness of the bladder, usually incontinence, less often retention; and some slight but constant sensory disturbances. Of these the two last-mentioned, some paræsthesia or anæsthesia, and early and constant implication of the bladder appear to be distinctive points, together with a very slow onset. The symptoms develop gradually and, as is so common in syphilitic nervous affections, they fluctuate or are transient. The last feature is well shown in one of two cases recorded by W. J. Dougherty,[22] in which transitory attacks of aphasia and impairment of the muscular power of the hands were observed. In the second case Romberg's symptom was well marked and the patient in walking kept his eyes fixed on the ground like a tabetic, but no inco-ordination was present. He also showed signs of advanced arteriosclerosis. In both these cases a careful examination of the cord was made after death and decided changes secondary to certain vascular lesions were found. In the first there was present an endarteritis of the vessels of the posterior roots and pia mater occupying a considerable vertical extent of the cord in the lower cervical and dorsal regions and resulting in a marginal sclerosis embracing in places its entire circumference, but especially involving the tract of Gowers, the direct cerebellar and the crossed pyramidal tracts. In one portion Goll's column was degenerated, and on examining the posterior roots they were observed in this region to contain scattered degenerated foci resulting from an obliterating endarteritis. The second case showed similar vascular lesions, but, instead of being scattered over a considerable vertical extent, they were more local and concentrated in a few segments only, so that the resulting sclerosis took the form of a localised transverse lesion with secondary ascending and descending tracts of degeneration. In both these cords the sclerotic zones were dense and showed few cells, a condition to be expected in chronic cases with slow onset and long duration such as these were, but in a case examined by Van Giesen within a year of onset the marginal zone was richly cellular, due apparently to active neuroglial proliferation. These findings of Dougherty, based, however, it must be remembered, on only two cases, are not in favour of regarding this form of spastic paralysis as a system disease. Rather the cord areas involved depend on the situation and extent of the vascular lesions, and in this way different types of the disease may be explained, such as those due to a marginal sclerosis, or those resembling a transverse myelitis, to a special form of which syphilitic spinal paralysis has by others been ascribed. The difficulty sometimes attending diagnosis is illustrated by a case shown by L. H. Mettler,[23] in which somewhat anomalous symptoms presented by a man, the child of two deaf mutes, who denied syphilis, were ascribed to multiple sclerosis or to syphilis of the spinal cord. *Hereditary spastic paraplegia*, unlike the form just described, is due not to an accidental condition acquired after birth, but to some inherent defect in the vitality of certain groups of neurons, whereby they are worn out in a few years, instead of lasting an average life-time. In this disease, which is to be distinguished from cases of congenital spastic paraplegia due to brain injury before or during birth, the stress of the early decay is first felt by the lateral columns, the posterior columns failing later and to a less extent. In *Friedreich's hereditary ataxia*, on the contrary, as Campbell Thomson[24] has recently pointed out in a clinical lecture upon the two diseases, the posterior columns are earliest and most affected, and hence arise the characteristic ataxic symptoms observed in the subjects of this family rather than hereditary disease, as Thomson prefers to call it. Special points on which he lays stress in the diagnosis of the latter besides the ataxy, family distribution, and early onset, are the slow nystagmus, very different from the quick jerky movements common in disseminated sclerosis, intact sensation, due to the fact that in the cord there are many paths for sensory impulses, and the deformities, lateral curvature of the spine, and the type of foot known as "pes cavus," in which the arch of the foot is accentuated and the toes cocked up like hammer-toes. Pes cavus may be the earliest sign observed in Friedreich's disease. The anatomical deformities are not found in *hereditary cerebellar ataxy*, which is also distinguished by exaggeration instead of loss of the deep reflexes and in which optic atrophy may appear. In hereditary spastic paraplegia, examples of which Thomson thinks are sufficiently numerous to warrant their description under a definite heading, the knee-jerks are also exaggerated. Trophic disturbances may occur here, but a deformity of the foot similar to that observed in Friedreich's ataxy is often the first complaint. The arching of the foot and toes only comes into play when the foot is raised from the ground, and a special splint has been devised by Thomson to remove the discomfort arising from the condition. It consists of a sole of firm leather fitted to the under-surface of the foot and pierced with suitable holes through which the toes can be laced into position by means of tapes. The device is simple and inexpensive and apparently effectual.

[22] Med. Rec., Aug. 5. [23] Jour. of Nerv. and Ment. Dis., April, p. 261. [24] Clin. Jour., June 14.

